# Bone Regeneration and Repair Materials

**DOI:** 10.3390/jfb15030078

**Published:** 2024-03-21

**Authors:** Marcio Mateus Beloti, Adalberto Luiz Rosa

**Affiliations:** Bone Research Lab, Ribeiraão Preto School of Dentistry, University of Saão Paulo, Ribeiraão Preto 14040-904, SP, Brazil; mmbeloti@usp.br

## 1. Introduction

Bone tissue has a remarkable ability to regenerate following injury and trauma [1,2,3]. However, the extent of bone loss or the presence of concurrent diseases can often surpass the regenerative ability, leading to the failure of conventional procedures and, consequently, the need for additional treatments [4,5]. In the field of bone repair, regenerative medicine encompasses all currently available treatments, including biological and material approaches and their combination, which are being evaluated by researchers and clinicians [6,7,8]. This Special Issue, entitled “Bone Regeneration and Repair Materials”, is composed of 14 original and 2 review articles that can be grouped into the following three categories: (1) physicochemical and mechanical characterizations of biomaterials for bone regeneration and implants, (2) strategies to induce bone repair using biomaterials and/or cells and (3) titanium (Ti) implants. We believe that this collection of information is of great interest to researchers and clinicians dealing with bone tissue and offers new insights into the interactions between bones and materials. We are appreciative to the *Journal of Functional Biomaterials* team for inviting us to Guest Edit this Special Issue and to the authors from eight countries who helped us build this impressive collection of scientific knowledge.

## 2. Overview of Published Articles

One of the main challenges in developing biomaterials as substitutes for bone tissue is the emulation of the physicochemical and mechanical properties of bones. Using nacre from mollusk shells with layered structures as a natural model for bio-inspired materials, Trabizian et al. (contribution 1) fabricated nacre-like composites of hydroxyapatite (HA) and polymers using a bidirectional freeze-casting technique. The mechanical characterization of the composites indicated that increasing the HA fraction enhanced the mineral bridge density, resulting in composites with higher flexural and compressive strengths, making them potential candidates for use in orthopedics, such as spinal fusion and bone fracture fixation implants. Based on the use of goose bone as a traditional medicine in Malay culture, Abdul Rahman et al. (contribution 2) described a method for preparing goose bone ash via bone calcination. They observed that sintering bones at 900 °C generated HA in the mineralogical phase with a calcium/phosphate atomic ratio of 1.64, which is very close to the ideal stoichiometric ratio of 1.67, creating possibilities for further investigations into therapeutic approaches using goose bone ash to repair bone tissue. Marine sponges have highly porous bodies and inorganic (biosilica) and collagen-like (spongin) organic contents, making them potential structures for use as natural scaffolds in bone tissue engineering [9]. Santos et al. (contribution 3) demonstrated that scaffolds produced from two species of marine sponges, despite exhibiting similar chemical compositions and porosities, presented distinct osteogenic potential when implanted in noncritical bone defects created in rat tibiae, with the scaffold with a higher degradation rate inducing more bone formation. As mentioned by the authors, the development of sustainable mariculture techniques is crucial for the generation of large-scale biomaterials derived from marine sponges for clinical therapeutic applications. With a focus on materials for bone tissue engineering, synthetic polymers with adequate printability and mechanical properties have been employed to fabricate scaffolds using several printing processing methods [10]. Gao et al. (contribution 4) applied digital light processing printing technology to fabricate a scaffold in which the addition of gelatin methacrylate enhanced the osteoblast differentiation of mesenchymal stem cells (MSCs) derived from rabbit bone marrow and increased bone formation in bone defects created in rabbit femurs. Among the biomaterials with potential to substitute bone tissue, 45S5 Bioglass^®^ is of great relevance as a synthetic glass that was found to chemically bond to bone [11,12]. Considering the repair of demanding bone defects such as the ones in osteoporotic bones, Araújo et al. (contribution 5) incorporated teriparatide, a recombinant fragment of the human parathyroid hormone, into 45S5 Bioglass^®^ and observed a promising result in terms of the bone repair of critical size defects created in ovariectomized rat calvariae. The association between biomaterials and cells is a smart approach that has been extensively investigated in bone tissue engineering [13,14]. For the first time, Adolpho et al. (contribution 6) combined photobiomodulation therapy, which is known to enhance bone repair, with a ceramic/polymer scaffold and MSCs and demonstrated that the association of these three tools increased the bone formation in rat calvarial defects, highlighting the need for innovative approaches and the combination of different techniques to regenerate large bone defects. Another potential therapeutical application of stem cells is in the prevention of the progressive degeneration of cartilage and subchondral bone triggered by temporomandibular disorders, a subject deeply explored in a bibliometric study performed by da Silva et al. (contribution 7). Oral health is directly linked to the integrity of mineralized tissues of the stomatognathic system, including bone, enamel and dentin, and biomaterials may contribute to the preservation of these tissues [15,16]. Dotta et al. (contribution 8) synthesized strontium-containing nanoparticles that formed a mineral layer and penetrated dentin tubules, which resisted an acidic environment and induced mineral deposition by human dental pulp stem cells. These nanoparticles combine the abrasive properties of calcium carbonate with the ability of strontium to induce mineralization and can be added to dentifrice formulations to treat dentin hypersensitivity. In the dental setting, implant placement in the posterior region of the maxilla often requires prior procedures, and sinus floor elevation is the most common surgical approach for oral implant-based rehabilitation [17]. Miyauchi et al. (contribution 9) used a rabbit model to compare the healing pattern after sinus floor lifting with either non-collagenated bovine or collagenated porcine xenografts, and they observed that despite both materials allowing bone formation, the collagenated xenograft underwent higher resorption, resulting in a greater amount of new bone. Despite the benefits of sinus floor elevation, Omori et al. (contribution 10) used a rabbit model to demonstrate that contact with grafts induced the thinning and possible perforation of the sinus mucosa, which have implications for clinical outcomes and need to be further investigated. Ti is a powerful tool for promoting oral rehabilitation because of its ability to osseointegrate, which might be affected by both bone quality and quantity and implant surface features [18,19]. Santiago et al. (contribution 11) developed and characterized a fluorapatite coating prepared using a hydrothermal method and deposited it on commercial Ti implants. In a rabbit tibia model, this surface promoted more bone formation, with increased bone-to-implant contact, compared to HA-coated implants, making fluorapatite coatings an interesting approach for the enhancement of implant osseointegration under challenging clinical conditions. Systemic diseases such as osteoporosis and hypertension may disrupt the process of Ti osseointegration [20,21]. Indeed, Mulinari-Santos et al. (contribution 12) worked with spontaneously hypertensive rats under antihypertensive therapy and showed that osteoporotic conditions induced by estrogen deficiency impaired Ti osseointegration, even when the implant surface was coated with an antiresorptive agent used to treat osteoporosis. Angiogenesis–osteogenesis coupling is crucial for Ti osseointegration, and obesity may disturb this circuit, disrupting bone–implant interactions [22]. Pinto et al. (contribution 13) established an in vitro experimental model of high adipogenesis and demonstrated that the proinflammatory environment created by obesity interferes with endothelial cell responses to a Ti-enriched medium, which could explain the high implant failure ratio in the obese population. To better understand bone response to Ti implants, it is important to investigate the cell signaling pathways involved in osseointegration [23,24]. Teixeira et al. (contribution 14) used a conditioned medium approach to show that a laser-modified Ti surface enhanced the osteoblast differentiation of MSCs by downregulating the Wnt signaling inhibitor, Dickkopf 1. Souza et al. (contribution 15) stated that the high osteogenic potential of a nanostructured Ti surface generated by chemical conditioning with H_2_SO_4_/H_2_O_2_ may be related to its capacity to regulate the Hedgehog and Notch signaling pathways, and that the activation of Hedgehog and the inhibition of Notch might synergistically affect osteoblast differentiation, especially in cells grown on nanotopography. Although Ti, ceramics, glasses, and polymers are the most commonly studied and used bone substitutes and implants, alternative materials have been investigated such as wood, which is a sustainable and renewable source suitable for the production of biomaterials using more environmentally friendly processes, a topic that is explored in a review prepared by Nefjodovs et al. (contribution 16).

## 3. Conclusions

This Special Issue demonstrates that a diverse range of materials and approaches have emerged as promising and powerful tools in regenerative medicine to promote bone repair, regeneration, and implant osseointegration. Additionally, this collection of studies sheds light on the need to understand the cellular mechanisms involved in the interactions between bones and materials in the search for optimized and smart therapies. We know that there is a long way to go before most of the strategies presented here can be implemented in clinical practice. Furthermore, we believe that the efforts of scientists and clinicians in both basic and translational fields will accelerate this ride and uncover innovative therapies to treat damaged bone tissue in a plethora of clinical situations, always seeking optimal patient well-being.
